# Calcification Characteristics of Low-Flow Low-Gradient Severe Aortic Stenosis in Patients Undergoing Transcatheter Aortic Valve Replacement

**DOI:** 10.1155/2015/802840

**Published:** 2015-09-07

**Authors:** Barbara E. Stähli, Thi Dan Linh Nguyen-Kim, Cathérine Gebhard, Thomas Frauenfelder, Felix C. Tanner, Fabian Nietlispach, Francesco Maisano, Volkmar Falk, Thomas F. Lüscher, Willibald Maier, Ronald K. Binder

**Affiliations:** ^1^University Heart Center, Department of Cardiology, University Hospital Zürich, 8091 Zürich, Switzerland; ^2^Institute of Diagnostic and Interventional Radiology, University Hospital Zürich, 8091 Zürich, Switzerland; ^3^University Heart Center, Department of Cardiovascular Surgery, University Hospital Zürich, 8091 Zürich, Switzerland

## Abstract

Low-flow low-gradient severe aortic stenosis (LFLGAS) is associated with worse outcomes. Aortic valve calcification patterns of LFLGAS as compared to non-LFLGAS have not yet been thoroughly assessed. 137 patients undergoing transcatheter aortic valve replacement (TAVR) with preprocedural multidetector computed tomography (MDCT) and postprocedural transthoracic echocardiography were enrolled. Calcification characteristics were assessed by MDCT both for the total aortic valve and separately for each leaflet. 34 patients had LFLGAS and 103 non-LFLGAS. Total aortic valve calcification volume (*p* < 0.001), mass (*p* < 0.001), and density (*p* = 0.004) were lower in LFLGAS as compared to non-LFLGAS patients. At 30-day follow-up, mean transaortic pressure gradients and more than mild paravalvular regurgitation did not differ between groups. In conclusion, LFLGAS and non-LFLGAS express different calcification patterns which, however, did not impact on device success after TAVR.

## 1. Introduction

Severe aortic stenosis (AS) is the most frequent valvular heart disease in the Western society [1, 2], and surgical aortic valve replacement (SAVR) has been the standard treatment once symptoms appear [3, 4]. However, treatment of high-risk patient subpopulations including those with low-flow low-gradient severe aortic stenosis (LFLGAS) remains challenging given their dismal prognosis when treated conservatively and the high surgical risk [5, 6]. Although SAVR has been associated with better clinical outcomes compared to medical management in LFLGAS [7, 8], the associated surgical risk may be prohibitive, and transcatheter aortic valve replacement (TAVR) has become a less invasive treatment option in high-risk patients [3, 9–11].

Aortic valve calcifications have extensively been studied in patients undergoing TAVR, and annular assessment is mostly based on multidetector computed tomography (MDCT) or transesophageal echocardiography [12, 13]. As degenerative aortic valves often display severe calcifications affecting the entire aortic root, a thorough preprocedural evaluation of annular dimensions and calcification characteristics is important for optimal device selection and transcatheter heart valve (THV) positioning, [14, 15]. However, there exists only few data about calcification patterns in patients with LFLGAS [16], and comparisons among different types of AS disease entities have not been performed yet.

The aim of this study was to assess aortic valve calcification characteristics in LFLGAS in comparison to non-LFLGAS patients and to delineate their impact on postprocedural THV function.

## 2. Materials and Methods

### 2.1. Patients and Procedures

Patients with symptomatic severe AS (mean transaortic systolic pressure gradient of ≥40 mmHg or an aortic valve area (AVA) of <1.0 cm^2^ or <0.6 cm^2^/m^2^) and both comprehensive baseline MDCT and postprocedural echocardiographic exams within 30 days were included in the analysis. Patients were then subdivided into two groups: (1) patients with LFLGAS (stroke volume index ≤35 mL/m^2^ and mean aortic valve gradient ≤40 mmHg), including patients with preserved (paradoxical LFLGAS) and reduced left ventricular ejection fraction (LVEF), and (2) patients with non-LFLGAS [17]. All patients were evaluated for TAVR by a multidisciplinary heart team.

Transthoracic echocardiography studies were performed at baseline and within 30 days after the procedure using commercially available equipment (Philips iE33, Philips Healthcare, Andover, MA, USA; GE Vivid 7, GE Healthcare, Milwaukee, WI, USA), and in accordance with the recommendations of the American Society of Echocardiography (ASE) and the European Association of Echocardiography (EAE) [18].

Transcatheter aortic valve procedures were performed between May 2008 and April 2012, either in the cardiac catheterization laboratory or the hybrid operation room, utilizing the Medtronic CoreValve (MCV; 26, 29, and 31 mm) or the Edwards SAPIEN prostheses (ES; 23, 26, and 29 mm) [19–21]. The study was approved by the local ethical committee, and informed written consent was obtained from all patients.

### 2.2. Multidetector Computed Tomography (MDCT) Image Acquisition and Analysis

Multidetector computed tomography (MDCT) with craniocaudal scan direction was performed using a second-generation 128-slice dual-source computed tomography (Somatom Definition Flash, Siemens Healthcare, Forchheim, Germany). A first bolus of 45 mL of Iopromide (Ultravist 300, 300 mg/mL, Bayer Schering Pharma, Berlin, Germany) was injected at a flow rate of 5 mL/sec, followed by a second bolus of 35 mL at a flow rate of 2.5 mL/sec and a bolus of 60 mL saline at the same flow rate. A signal attenuation threshold of 100 Hounsfield units (HU) was used for bolus tracking in the ascending aorta. The scan ranged from the apex of the lungs to the symphysis and was started automatically based on the previous 10 heart beats aiming at a 60% RR-interval at the level of the sinotubular junction.

**Figure 1:**
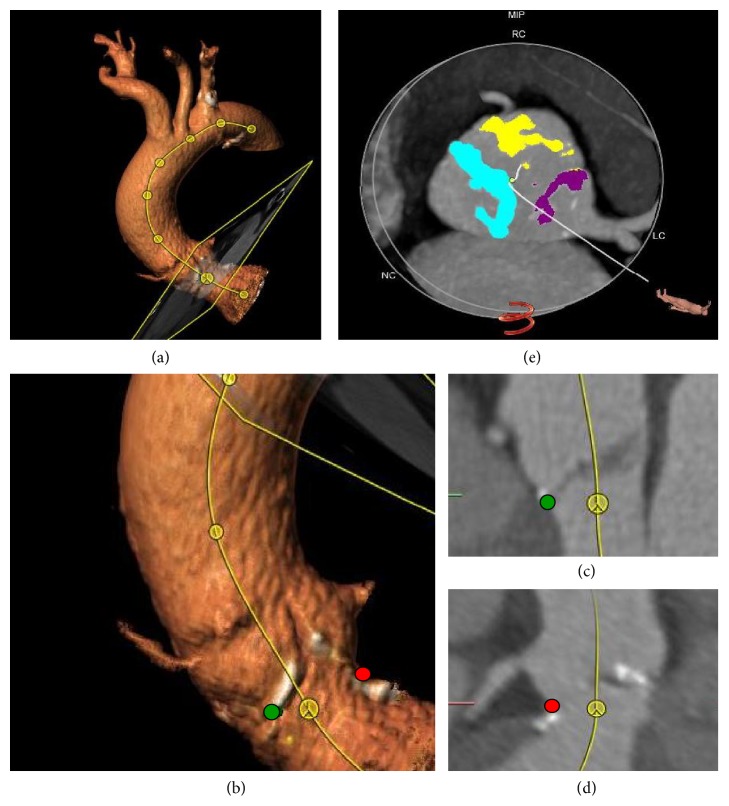
Multidetector computed tomography (MDCT) analysis illustrating calcification measurements. (a) Centerline of the aortic root and the ascending aorta. (b), (c), and (d) Anchor points at the level of the insertion of the aortic leaflets. (e) Measurement of calcification volume and mass.

As previously described, a dedicated software (3mensio Structural Heart 6.0, Bilthoven, Netherlands) was utilized for quantitative image analysis [20]. First, the centerline of the aortic root and the ascending aorta was drawn semiautomatically. Then, the aortic annular plane was defined at the insertion of the aortic leaflets. Calcification characteristics, including calcification volume, mass, and density (Hounsfield units, HU), were assessed semiautomatically for the total aortic valve as well as separately for each leaflet [20]. To determine aortic valve calcification asymmetry, the maximal difference of both calcification mass and volume between the three aortic cups was calculated (Figure 1). The aortic annular eccentricity index was calculated as 1 − diameter_minimal_/diameter_maximal_ as previously described [22]. Multidetector computed tomography images were analyzed by experienced imaging specialists who were blinded to echocardiographic and clinical data.

### 2.3. Statistical Analysis

Continuous variables are presented as means ± standard errors and categorical variables as frequencies and percentages. Normality distribution was tested with the Shapiro Wilk test and homogeneity of variance with the Levene test, respectively. Continuous variables were tested for differences with the unpaired Student *t*-test or the Mann-Whitney *U* test and categorical variables with the Pearson *χ* ^2^ test or the Fisher exact test as appropriate. Correlations between two variables were specified by the Spearman correlation coefficient. All tests were 2-tailed, and a *p* value of <0.05 was established as the level of statistical significance for all tests. All statistical analyses were performed using IBM-SPSS version 21 (IBM Corp.) for Windows.

## 3. Results

### 3.1. Baseline Characteristics

Thirty-four (25%) patients had LFLGAS and 103 (75%) non-LFLGAS, respectively. Out of the 34 patients with LFLGAS, 18 (53%) patients had preserved LVEF >50% (paradoxical LFLGAS). All patients were at increased surgical risk as expressed by a mean Society of Thoracic Surgeons Predicted Risk of Mortality (STS-PROM) score of 5.6 ± 0.3%. Peripheral vascular disease and atrial fibrillation were more frequently observed in LFLGAS patients (Table 1).

Mean aortic valve gradient was 29 ± 1.2 mmHg in LFLGAS patients and 55 ± 1.4 mmHg in non-LFLGAS patients (*p* < 0.001), and stroke volume index was 27 ± 0.9 mL/m^2^ and 32 ± 1.1 mL/m^2^ in both groups (*p* = 0.009). Aortic valve area was 0.81 ± 0.04 cm^2^ in LFLGAS patients and 0.69 ± 0.02 cm^2^ in non-LFLGAS patients, respectively (*p* = 0.004).

### 3.2. Procedural Characteristics

Transfemoral procedures were performed in 26 (77%) LFLGAS and in 84 (82%) non-LFLGAS patients (*p* = 0.62), and the MCV prosthesis was utilized in 19 (56%) LFLGAS and in 49 (48%) non-LFLGAS patients, respectively (*p* = 0.43). Device success at 72 hours was achieved in 31 (91%) patients in the LFLGAS group and in 92 (89%) patients in the non-LFLGAS group (*p* = 1.0). Failed device success was due to more than mild aortic regurgitation in 13 patients and due to an increased mean transvalvular pressure gradient >20 mmHg in 1 patient.

### 3.3. Aortic Annulus Dimensions Assessed by MDCT

Annulus area and perimeter were 5.04 ± 0.19 cm^2^ and 81.1 ± 1.5 mm in patients with LFLGAS and 4.86 ± 0.09 cm^2^ and 79.7 ± 0.8 in those with non-LFLGAS, without any differences between groups (*p* = 0.31 for annulus area and *p* = 0.36 for annulus perimeter). Eccentricity index was similar in both groups (0.22 ± 0.01 for LFLGAS and 0.19 ± 0.01 for non-LFLGAS; *p* = 0.08).

### 3.4. Aortic Valve Calcification Pattern Assessed by MDCT

In the total patient cohort, aortic valves were severely calcified with a total aortic valve calcification volume of 1106 ± 164 mm^3^, a total aortic valve calcification mass of 660 ± 34 mg, and a total aortic valve calcification density of 817 ± 10 HU. Total aortic valve calcification volume was 627 ± 61 mm^3^ in LFLGAS versus 1264 ± 216 mm^3^ in non-LFLGAS (*p* < 0.001) and total aortic valve calcification mass was 398 ± 39 mg in LFLGAS versus 747 ± 41 mg in non-LFLGAS (*p* < 0.001, Table 2). Total aortic valve calcification density was lower in LFLGAS (766 ± 18 HU) compared to non-LFLGAS (834 ± 12 HU; *p* = 0.004). Regarding each leaflet separately, calcification characteristics differed significantly for the noncoronary, the left, and the right coronary cusps, with lower values for LFLGAS compared to non-LFLGAS (Table 2).

After the exclusion of patients with preserved LVEF >50% (paradoxical LFLGAS), total aortic valve calcification volume was 710 ± 107 mm^3^ and 1264 ± 216 mm^3^ in true LFLGAS and non-LFLGAS patients (*p* = 0.01); total aortic valve calcification mass was 449 ± 69 mg and 747 ± 41 mg (*p* = 0.005) and total aortic valve calcification density 753 ± 28 HU and 834 ± 12 HU (*p* = 0.02), respectively. In LFLGAS patients matched for AVA to 34 non-LFLGAS patients, similar trends were observed, without, however, reaching statistical significance. In the matched patient cohort, total aortic valve calcification volume was 643 ± 102 mm^3^ and 989 ± 145 mm^3^ in LFLGAS and non-LFLGAS patients (*p* = 0.07); total aortic valve calcification mass was 398 ± 61 mg and 677 ± 105 mg (*p* = 0.06) and total aortic valve calcification density 755 ± 20 HU and 803 ± 27 HU (*p* = 0.16), respectively.

Maximal differences of calcification volumes and masses between the three aortic cusps, a measure of calcification asymmetry, were lower in LFLGAS (162.9 ± 16.8 mm^3^ and 168.2 ± 59.0 mg) compared to non-LFLGAS patients (286.6 ± 21.9 mm^3^ and 234.2 ± 18.3 mg; *p* < 0.001).

Baseline mean aortic valve gradients correlated significantly with aortic valve calcification volume (*r* = 0.42, *p* ≤ 0.001), mass (*r* = 0.46, *p* ≤ 0.001), and density (*r* = 0.29, *p* = 0.001).

### 3.5. Symptomatic and Hemodynamic Improvement at 30 Days

**Figure 2:**
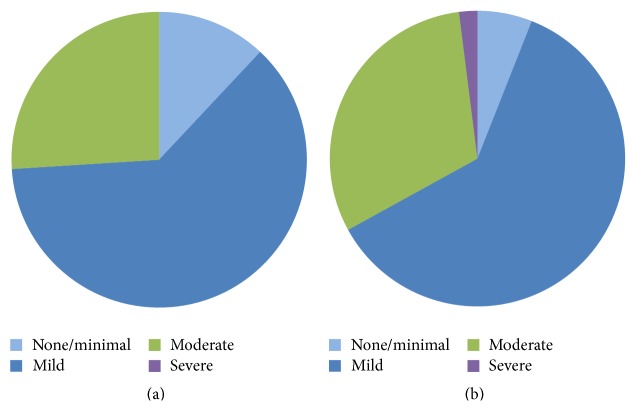
Postprocedural paravalvular aortic regurgitation in patients with low-flow low-gradient severe aortic stenosis (LFLGAS) and those with non-LFLGAS. (a) LFLGAS patients. (b) Non-LFLGAS patients.

At 30-day follow-up, mean transaortic pressure gradient was 8.8 ± 05 mmHg in LFLGAS patients and 9.3 ± 0.5 mmHg in non-LFLGAS patients (*p* = 0.58). More than mild paravalvular regurgitation at 30 days did not differ between groups (Figure 2). In the LFLGAS group, 9/34 (26%) patients had more than mild paravalvular regurgitation compared to 34/103 (33%) patients in the non-LFLGAS group (*p* = 0.52).

In both the LFLGAS and the non-LFLGAS group, patients with more than mild paravalvular regurgitation at 30 days tended to have higher total aortic valve calcification volume (708 ± 167 mm^3^ versus 598 ± 60 mm^3^, *p* = 0.67 for LFLGAS, and 1758 ± 637 mm^3^ versus 1021 ± 69 mm^3^, *p* = 0.29 for non-LFLGAS), mass (484 ± 110 mg versus 367 ± 34 mg, *p* = 0.38 for LFLGAS, and 836 ± 77 mg versus 703 ± 47 mg, *p* = 0.17 for non-LFLGAS), and density (818 ± 40 HU versus 747 ± 18 HU, *p* = 0.07 for LFLGAS, and 867 ± 24 HU versus 817 ± 12 HU, *p* = 0.10 for non-LFLGAS), albeit without reaching statistical significance.

**Figure 3:**
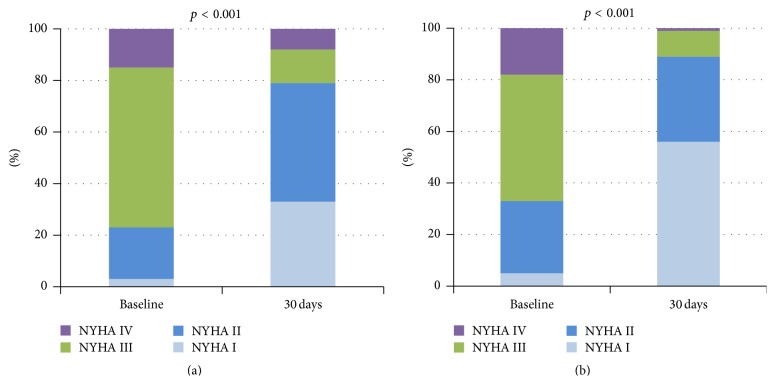
Postprocedural symptomatic improvement in patients with low-flow low-gradient severe aortic stenosis (LFLGAS) and those with non-LFLGAS. (a) LFLGAS patients. (b) Non-LFLGAS patients. NYHA indicates New York Heart Association functional class. *p* ≤ 0.05 denotes a significant difference between patients with low-flow low-gradient severe aortic stenosis (LFLGAS) and those with non-LFLGAS.

At baseline, 26/34 (77%) and 69/103 (67%) patients were in New York Heart Association (NYHA) functional class III or IV. A significant symptomatic improvement was observed in both groups, with 5/24 (21%) LFLGAS patients and 8/73 (11%) non-LFLGAS patients in NYHA functional class III or IV at 30-day follow-up (*p* < 0.001; Figure 3).

## 4. Discussion

This study demonstrates that calcification characteristics differ between LFLGAS and non-LFLGAS patients, with less calcification mass, volume, and asymmetry in the former compared to the latter. In both groups, a substantial symptomatic and hemodynamic benefit after TAVR was observed with comparable postprocedural THV function.

### 4.1. Low-Flow Low-Gradient Severe Aortic Stenosis

LFLGAS is considered as distinct clinical entity, and the observed prevalence of 25% in our patient cohort is in line with previously reported data [8, 17]. In the era of emerging transcatheter approaches, the optimal therapeutic strategy still remains unclear. Substantial clinical benefits have recently been reported in LFLGAS patients undergoing TAVR [23, 24], with reduced long-term mortality when compared to medical management alone [24]. However, in this patient subgroup, clinical outcomes following TAVR have not extensively been studied yet, and inconsistencies do exist with regard to benefits in comparison to high-gradient AS. While low transaortic pressure gradients have clearly been associated with an increased mortality in patients undergoing TAVR [23, 25–27], a recent study reported comparable postprocedural short- and long-term outcomes in patients with LFLGAS and those with high-gradient AS, most interestingly, irrespective of left ventricular systolic function [28]. In line with these findings, similar outcomes after TAVR have been reported in patients with low-gradient severe AS and preserved LVEF and those with high-gradient severe AS [29]. Of note, low-flow states, and not impaired left ventricular systolic function or low transvalvular gradients, have mainly been suggested to add to the increased risk associated with this challenging patients' subset [24, 30]. Hence, optimal postprocedural therapy and minimization of postprocedural complications including paravalvular regurgitation are of utmost importance in this patient population to further reduce cardiovascular morbidity and mortality [28].

### 4.2. Importance of Aortic Valve Calcifications in THV

Preprocedural evaluation of aortic root characteristics is important for procedure planning and often remains challenging given the complex three-dimensional annular structure [31]. Mostly, preprocedural assessment is based on multimodality imaging including transthoracic and transesophageal echocardiography as well as MDCT [32–36], with MDCT being considered the imaging modality which provides the most accurate measures of annular dimensions and calcifications [14, 37, 38]. In particular, utilizing MDCT for the evaluation of calcifications, qualitative measurements of different calcification characteristics become possible compared to a rather qualitative echocardiographic evaluation. In both patient subgroups, a trend towards more severe aortic valve calcifications in patients with more than mild paravalvular regurgitation was observed, pointing towards the association between annular calcifications and paravalvular regurgitation after TAVR irrespective of AS disease entity.

As expected, aortic valves were highly calcified in this patient cohort. Interestingly, patients with LFLGAS displayed less severe aortic valve calcifications compared to those with non-LFLGAS. These findings suggest that annular structures may be unequally affected by fibrotic and/or calcific changes in different AS disease entities. Indeed, low aortic valve gradients have been associated with increased myocardial fibrosis in biopsy samples and more late-gadolinium enhancement in magnetic resonance imaging studies [39]. Further, as baseline mean aortic valve gradients correlated significantly with aortic valve calcification severity in this patient cohort, a cause-effect relationship between mechanical forces and aortic valve calcifications may be hypothesized, and adverse patterns of fluid shear stress may promote calcification processes. This interpretation is supported by recent studies identifying baseline hemodynamic aortic stenosis severity as an independent predictor of disease progression [40, 41]. Hence, altered mechanical forces associated with lower transvalvular pressure gradients could at least in part explain lower degrees of valvular calcifications observed in LFLGAS patients. As AVA was larger in LFLGAS patients, some have speculated that this disease entity represents a precursor of high-gradient aortic stenosis. However, the fact that similar trends towards higher calcifications in non-LFLGAS patients were observed in patients matched for AVA suggests that possible differences in AS severity as assessed by AVA may not fully explain the different degree of valvular calcifications observed among groups. However, we cannot exclude completely that slightly different AS severities as reflected by significantly different AVA between LFLGAS and non-LFLGAS patients may at least in part have contributed to the observed findings. However, pathophysiological considerations remain speculative in this context, and further large-scale clinical studies are needed to elucidate key pathological mechanisms underlying these observations.

### 4.3. Study Limitations

There are some limitations that need to be considered such as the single center design, the retrospective nature, and the rather small patient population. In particular, larger studies are needed to allow for comparisons among different LFLGAS subgroups and to elucidate the association between calcifications and paravalvular regurgitation in LFLGAS and non-LFLGAS patients. In addition, the comparison of patients with and without flow reserve was precluded, as dobutamine stress echocardiography was not performed in the entire patient cohort. This study was designed to assess calcification characteristics and their impact on postprocedural THV function and, thus, included patients with preprocedural MDCT and postprocedural echocardiography within 30 days, a design which did not allow for a comparison of mortality rates among groups.

## 5. Conclusion

This study demonstrates that aortic valve calcification characteristics vary between patients with LFLGAS and non-LFLGAS with less valvular calcification mass, volume, and asymmetry observed in the former. These observations evidence the unmet need for further characterization and stratification of this challenging AS patients' subgroup aiming at an enhanced understanding of different AS disease entities. Importantly, substantial symptomatic and hemodynamic benefit was observed in both patient groups with excellent postprocedural THV function.

## Figures and Tables

**Table 1:** Baseline characteristics.

Baseline characteristics	Combined	LFLGAS	Non-LFLGAS	*p* value
Age, years	83 ± 0.5	81 ± 1.2	83 ± 0.5	0.07
Male	73 (53)	23 (68)	50 (49)	0.07
Coronary artery disease	91 (66)	25 (74)	66 (64)	0.40
Previous CABG	30 (22)	11 (32)	19 (18)	0.10
Diabetes mellitus	36 (26)	11 (32)	25 (24)	0.37
Hypertension	110 (80)	25 (74)	85 (83)	0.32
Cerebrovascular disease	37 (27)	7 (21)	30 (29)	0.38
Chronic obstructive pulmonary disease	21 (15)	5 (15)	16 (16)	1.00
Peripheral vascular disease	37 (27)	15 (44)	22 (21)	**0.01**
Pulmonary hypertension	19 (14)	7 (21)	12 (12)	0.25
Atrial fibrillation	35 (26)	14 (41)	21 (20)	**0.02**
Porcelain aorta	16 (12)	5 (15)	11 (11)	0.54
Creatinine (*µ*mol/L)	117 ± 5	112 ± 6	133 ± 3	0.14
STS-PROM score (%)	5.6 ± 0.3	5.3 ± 0.30	6.3 ± 0.7	0.24
LVEF (%)	55 ± 1.1	48 ± 2.7	57 ± 1.1	**0.003**
Mean aortic valve gradient (mmHg)	48 ± 1.4	29 ± 1.2	55 ± 1.4	**<0.001**
Stroke volume index (mL/m^2^)	31 ± 0.9	27 ± 0.9	32 ± 1.1	**0.009**
Aortic valve area (cm^2^)	0.72 ± 0.02	0.81 ± 0.04	0.69 ± 0.02	**0.004**

CABG: coronary artery bypass grafting, STS-PROM: Society of Thoracic Surgeons Predicted Risk of Mortality, and LVEF: left ventricular ejection fraction, respectively. Results are presented as mean and standard error, or as number and percentages. *p* ≤ 0.05 denotes a significant difference between patients with low-flow low-gradient severe aortic stenosis (LFLGAS) and those with non-LFLGAS.

**Table 2:** Aortic valve calcification characteristics.

Parameters	Combined	LFLGAS	Non-LFLGAS	*p* value
Total aortic valve				
Calcification volume (mm^3^)	1106 ± 164	627 ± 61	1264 ± 216	**<0.001**
Calcification mass (mg)	660 ± 34	398 ± 39	747 ± 41	**<0.001**
Calcification density (HU)	817 ± 10	766 ± 18	834 ± 12	** 0.004**
Noncoronary cusp				
Calcification volume (mm^3^)	378 ± 20	222 ± 20	429 ± 24	**<0.001**
Calcification mass (mg)	282 ± 20	200 ± 60	309 ± 18	**<0.001**
Calcification density (HU)	810 ± 13	754 ± 20	852 ± 11	** 0.01**
Right coronary cusp				
Calcification volume (mm^3^)	272 ± 18	201 ± 26	295 ± 21	0.06
Calcification mass (mg)	186 ± 13	127 ± 17	206 ± 16	** 0.03**
Calcification density (HU)	801 ± 10	752 ± 19	829 ± 16	** 0.004**
Left coronary cusp				
Calcification volume (mm^3^)	309 ± 22	204 ± 28	344 ± 27	** 0.01**
Calcification mass (mg)	219 ± 17	127 ± 18	250 ± 21	**<0.001**
Calcification density (HU)	835 ± 10	785 ± 17	817 ± 11	** 0.003**

*p* ≤ 0.05 denotes a significant difference between patients with low-flow low-gradient severe aortic stenosis (LFLGAS) and those with non-LFLGAS.
